# Psychosocial factors play a central role in determining SNAP utilization for farm workforce

**DOI:** 10.3389/fpubh.2024.1402142

**Published:** 2024-07-31

**Authors:** Briana E. Rockler, Stephanie K. Grutzmacher, Jonathan Garcia, Ellen Smit, Marc Braverman

**Affiliations:** ^1^Public Health and Environmental Studies, College of Arts and Sciences, University of Wisconsin–Eau Claire, Eau Claire, WI, United States; ^2^College of Health, Oregon State University, Corvallis, OR, United States

**Keywords:** SNAP, federal safety net, farm workforce, farmworker, food insecurity, Andersen Behavioral Model

## Abstract

**Introduction:**

Federal food safety net programs, like the Supplemental Nutrition Assistance Program (SNAP), may not reach vulnerable populations like rural residents, immigrants, and Latinx individuals. Because these groups are overrepresented among the farm workforce, exploring SNAP utilization among farm communities may clarify the role it plays in alleviating food insecurity.

**Methods:**

In-depth interviews were conducted with 31 farmworkers and farm owners. Patterns and predictors of SNAP utilization were organized using an adapted Andersen Behavioral Model of Health Service Utilization.

**Results:**

Psychosocial factors played the central role in participants’ use of SNAP. Discussion: Opportunities to improve the design and delivery of SNAP include expanded eligibility cut-offs and targeted engagement mechanisms.

## 1 Introduction

Policies and programs that effectively target food insecurity are critical in ensuring access to healthful food for all U.S. residents. The most extensive federal food safety net program, the Supplemental Nutrition Assistance Program (SNAP), is designed to increase low-income households’ food purchasing power. According to United States Department of Agriculture (USDA) data, SNAP served 78 percent of all eligible individuals in 2020 before the pandemic (1). While such safety net resources are ostensibly available to all eligible individuals, utilization rates differ across diverse sectors of the eligible population, revealing distinctly vulnerable subgroups (2), namely, Hispanic/Latinx households, those living outside of metropolitan areas, older adults without dependents (3), and both non-citizen and naturalized immigrant households (4). Tailored engagement of specific racial, age, and geographic groups may augment the food safety net’s reach. Engagement must be informed by evidence to focus on underserved communities’ unique needs and the factors that drive them to utilize available programs. In this study, we explore SNAP utilization through the experiences of a particularly important vulnerable subgroup, the farm workforce.

Residents of rural agricultural communities face higher rates of food insecurity than those who reside in urban areas. Living in a rural setting presents unique challenges that may lead to inadequate access to food at home, including geographical isolation, limited job opportunities, low availability of household resources, and limited access to food retailers (5–7). In addition, those individuals who work on a farm, including farm owners and farmworkers, encounter added risks to their health and well-being due to the environmental, financial, and social factors that are characteristic of farm labor (8). High rates of diet related chronic disease, stress, injury, mental illness, substance abuse, and suicide among the farm workforce and rural communities highlight the need for policies intended to improve human health and well-being for farm owners, farmworkers, and their families (5, 9). High prevalence of food insecurity has been documented among farmworkers, and Latinx farmworkers encounter even greater rates of food insecurity (7, 10–16). Safety net programs that provide food access, like SNAP, are designed to enhance household resources to obtain food and improve nutrition. As such, participation in SNAP may address diet-related chronic disease and food insecurity jointly (17). Although SNAP utilization may enable farmworker and farm owner access to food, their eligibility for and access to the program may be limited (18–20).

According to federal guidelines, only U.S. citizens and qualified noncitizens can participate in SNAP. Eligible noncitizens include lawfully present immigrant children, refugees, asylees, and qualified legal immigrant adults living in the U.S. for a minimum of 5 years. There are also specific income, asset, age, disability, work, and time-limit eligibility criteria that can vary between states (21). Both farmworkers and farm owners with economic hardship may find themselves ineligible for SNAP. While undocumented farmworkers and those who have held lawful worker status for less than 5 years are not eligible to participate, many farmworker households are eligible to receive SNAP for children or other household members. SNAP utilization is low among all Hispanic/Latinx farmworkers (including those that are citizens, lawfully present, and undocumented) compared to non-Hispanic/Latinx White farmworkers (19), despite SNAP’s potential to improve racial health disparities experienced by farmworkers (22). Although farm owners face fewer eligibility restrictions, seasonal and market-dependent income fluctuations can result in periods of eligibility and ineligibility throughout the year (23). Self-employed farm owners must also provide proof of net income, which can be complicated and time-consuming to verify and is prone to calculation errors (23, 24). SNAP-eligible farm owners and farmworkers may not utilize the program for a variety of reasons, including lack of program awareness, lack of access to be able to apply and recertify for benefits, confusion about eligibility status, stigma related to utilization, fear of immigration consequences, benefit inadequacy, among others (25–27).

Previous studies have broadly identified predictors of SNAP utilization (28–30). The most consistent and robust predictor of SNAP utilization is poverty status. In line with income-based eligibility guidelines, this includes households with gross monthly income less than 130% of the federal poverty level (30). Strickhouser Vega et al. (30) used their adapted version of the Andersen Behavioral Model of Health Service Utilization (31) to confirm the following predictors of SNAP utilization among the general population: females, under 35, non-Hispanic/Latinx Black, Hispanic/Latinx, not married, households with kids or a disabled family member, less than high school education, and those with higher perceived food need. These attributes largely mirror the demographic profiles included in the SNAP annual program reports, consistently showing the highest utilization rates among non-Hispanic/Latinx Black and Hispanic/Latinx households and lower utilization among eligible adults over age 60 and the working poor (1, 32). Still, little is known about the factors that drive SNAP utilization among vulnerable populations with low program utilization rates, like farmworkers and farm owners.

To build upon the existing knowledge that predicts SNAP utilization among the general population this study uses the Andersen model as a framework to better understand the issues that lead to low SNAP utilization among the farm workforce (31). We adapt and propose a new theoretical model that depicts the patterns of SNAP utilization and considers the unique vulnerabilities encountered by farmworkers and farm owners. In doing so, we aim to identify the factors that drive SNAP utilization among farming families. Study findings may clarify the role of SNAP within farm communities to inform tailored engagement mechanisms and identify opportunities for policy improvement.

## 2 Materials and methods

### 2.1 Conceptual framework

The Andersen Behavioral Model of Health Service Utilization has been widely used to study health services and adapted to analyze SNAP use (30, 33). According to the model, utilization of health care services can be predicted by predisposing, enabling, and need factors. The framework evaluates access (i.e., equity, efficacy, efficiency) and the environmental conditions that impact access and use. According to Andersen (31), the model reveals equity of access to a health service. A signal of equity is when access is driven strictly by demographic characteristics, whereas access moderated by psychosocial and enabling resources is a signal of inequity. As the purpose of the current study was to understand low SNAP utilization, the Andersen framework enabled the qualitative exploration of issues related to SNAP access and equity. Acknowledging that the original model may not fully capture the psychosocial factors that determine service access and equity, Bradley et al. (34) expanded the Andersen model in their study on the role of race and ethnicity in health service utilization. When aggregated, the original and expanded Andersen models provide a comprehensive framework to organize the demographic, social, and situational factors and domains that drive SNAP utilization. Predisposing factors are the sociodemographic characteristics that influence an individual or household’s likeliness to participate in SNAP (31). Psychosocial factors are those that influence decision-making related to the intended behavior. Derived from the Theory of Planned Behavior (35), the model incorporates four psychosocial domains: attitudes, knowledge, subjective norms, and perceived control (34). Enabling factors are those community and policy-level resources necessary for SNAP utilization. Need factors are individual perceptions of household needs. Finally, use factors include the perceived value and experiences of SNAP utilization.

### 2.2 Study design

This qualitative study of farmworkers and farm owners (*N* = 31) in Oregon uses a modified grounded theory approach (36) to create a description of SNAP use among the farm workforce that was grounded in or emerged from interview data. A modified grounded theory methodology uses an inductive approach to build evidence and a deductive process to make predictions about other experiences (37). Credibility, dependability, and confirmability strategies were integrated into the study design to promote rigor and trustworthiness using established quality criteria (38). Methods included time sampling, repetition and reframing of questions, reflexivity assessments, and dense descriptions of each interview (38, 39).

### 2.3 Sample

Using convenience sampling methods, individuals from the farm workforce who may experience food insecurity or who work with or employ others who experience food insecurity were recruited to participate in in-depth, face-to-face, semi-structured interviews. Farmworkers were recruited from a housing complex and an orchard in March and April 2018. At the housing complex, a staff member recruited using informational flyers, and the study team recruited on-site for same-day interviews. Participants interviewed at the housing complex were employed on varying surrounding farms. On the orchard, the owners provided recruitment materials to employees to participate. The housing complex and orchard were situated in one rural community located in a region of the state known for its productive orchards, primarily pears, apples, and cherries.

Farm owners were recruited from local direct-to-consumer markets, Cooperative Extension contact lists, or Extension Agent nomination to participate in interviews between December 2019 and March 2020. Farms were situated in various rural regions (>10 miles from a population center for 40,000 people or greater) throughout Oregon spanning from the Columbia River Gorge to the southern Willamette Valley. As Extension staff had a reputable presence within rural farm communities, their involvement fostered credibility and trustworthiness among participants who may have been otherwise reluctant to participate in research.

### 2.4 Measures

Semi-structured interview guides were designed to capture participant experiences managing food hardship as a central phenomenon (40). The interview guide for farmworkers was developed over one-year using stakeholder input and a review of both gaps in the literature and published instruments (41). It contained 51 multi-part questions covering three topical domains: food security status – 7 questions (i.e., “Are there times of the month or times of the year when food is harder for you to get?”), perceptions of food access and available resources – 30 questions (i.e., “What do you do once you start to run out of food?” “To what extent do you think the resources available to you help or hurt your ability to make ends meet?”), and food safety net utilization – 14 questions (i.e., “Do you participate in SNAP? Why or why not?”). One member of the research team translated the instrument into Spanish, another member translated it back into English, and the final Spanish translation was checked for accuracy.

The interview guide for farm owners was derived from the farmworker interview guide with additional questions added after conferring with faculty from Cooperative Extension and a review of gaps in the literature. The farm owner guide excluded components related explicitly to the farmworker role and included questions about the farm ownership role. Questions that prompted farm owners to talk about their financial challenges and hardships were drawn from interview-based research with farm owners in Oregon (42). It contained 49 multi-part questions covering four topical domains: farm owner food security status – 13 questions (i.e., “Do you ever feel worried, stressed, or sad about getting enough food for yourself and your family?”), farm characteristics – 16 questions (i.e., “Please describe your farm operation: What do you produce? What size is your farm? Who works on your farm?” “What are your beliefs about the farming you do?”), farmworker food security – 12 questions (i.e., “Have your workers come to you for food because they worry about having enough?”), community food security – 5 questions (i.e., “What role do you think you or your farm business play in ensuring food security in the broader community?”), and the food system – 3 questions (i.e., “What changes would you like to see in the food system that would help more people be food secure?”).

For both interview guides, questions were iteratively redeveloped and refined in consideration of emerging themes, evolving perspectives, and the positionality of the researchers regarding the influence of their roles, attitudes, and biases in the inquiry process as community outsiders (40, 43, 44). Demographic information, including race/ethnicity, age, sex, household size, annual income, role on the farm, farm type, other work or jobs they have, and how many days per week they typically work, was reported by participants verbally in response to prompts. The USDA Household Food Security Survey Module (HFSSM) 6-item Short Form was selected as a descriptive measure to screen for food insecurity among the participants.

### 2.5 Procedures

Interviews were conducted in Spanish or English, depending on the preference of the interviewee. Informed consent was obtained verbally from farmworkers and in written form from farm owners before their interview. Interviews ranged between 40 and 159 min. Each participant received $25 cash for their time, and all were offered a snack. Interviews were conducted in spaces convenient for the participants, including on the farm, in farm owners’ homes, community meeting spaces, and Extension offices. The current study was conducted as part of a broader research project that aimed to investigate the ways in which farmworkers and farm owners utilize formal and informal food safety nets to cope with hardship. The Oregon State University Institutional Review Board approved study procedures.

### 2.6 Data collection

Interviews were digitally recorded then transcribed verbatim in the language of recording by a professional transcription service. The transcription service then translated Spanish language interviews into English. Participants were encouraged to share any experiences or ideas inspired by the interview questions (40). Interviewers were trained to use follow-up questions to pursue emerging theoretical constructs, practices, relevant terminology, and insights (45).

### 2.7 Analysis

A priori code families designated (1) SNAP utilization, (2) individual values and beliefs as they relate to SNAP, and (3) sociocultural and community assets and vulnerabilities (46). Emergent codes were developed as they occurred in the data and then organized by factor, domain, and theme adapted from the original and expanded Andersen models. Using MAXQDA, data were coded in three stages. First, the primary author open-coded interview transcripts to identify and sort concepts. Second, the primary and senior authors convened over several sessions to review, consider alternatives and agree upon codes. Axial coding followed to organize related themes, allowing for a deeper analysis of the process that determined SNAP utilization. Themes emerged around cognitive, affective, and evaluative processes related to the intent to use SNAP. Some themes were interrelated, overlapping within and between categorical domains, and each domain was linked by pathways of influence, revealing a process through which the intent to use SNAP progressed. Data were not mutually exclusive to one factor or domain and could have been coded under multiple domains. In the final step, the primary author used selective coding to develop a theoretical model that depicted the factors contributing to SNAP utilization among the farm workforce (47).

## 3 Results

### 3.1 Sample characteristics

The sample included farmworkers (*n* = 18) and farm owners (*n* = 13) in Oregon aged 30–75 years old (*M* = 49, SD = 13.3). All farmworkers identified as Hispanic or Latinx. Farm owners identified as non-Hispanic/Latinx White (*n* = 11) or non-Hispanic/Latinx Asian (*n* = 2). Most participants were born outside of the U.S. (61%). The preferred language of their interview was distinctly divided between Spanish language interviews for farmworkers and English language interviews for farm owners. Eighty-seven percent said that they have access to adequate quantities of food, but less (71%) reported that their food preferences were met. Over half were food secure (58%) as evaluated by the USDA HFSSM. Twenty-six percent were currently utilizing SNAP, 32% had previously used SNAP, and 42% had never used SNAP. In response to a prompt regarding their income level, a quarter of participants reported income <130% of the federal poverty level which would make them income-eligible for SNAP. Further descriptive characteristics of the participants can be found in Table 1.

**Table 1:** Participant characteristics by SNAP program utilization (*N* = 31), Oregon, USA.

Factors	Sample total *N* = 31 (col. %)	Current SNAP utilization *n* = 8 (row %)	Not currently using SNAP *n* = 23 (row %)
**Predisposing and psychosocial**			
Farmworker	18 (58)	5 (28)	13 (72)
Farm owner	13 (42)	3 (23)	10 (77)
Age			
<35	3 (10)	0 (0)	3 (100)
35–49	13 (42)	3 (23)	10 (77)
50–59	8 (26)	2 (25)	6 (75)
60+^a^	7 (22)	3 (43)	4 (57)
Sex			
Female	16 (52)	2 (13)	14 (88)
Male	15 (48)	6 (40)	9 (60)
Race/Ethnicity			
Hispanic/Latinx	18 (58)	6 (40)	9 (60)
White, Non-Hispanic/Latinx	11 (36)	2 (14)	12 (86)
Asian, Non-Hispanic/Latinx	2 (6)	0 (0)	2 (100)
Born in the U.S.	12 (39)	2 (17)	10 (83)
Marital status			
Yes, living together in U.S.	21 (68)	8 (38)	13 (62)
No	10 (32)	0 (0)	7 (100)
Household size (people in the home)			
1	3 (10)	0 (0)	3 (100)
2–3	21 (68)	6 (29)	15 (71)
4+	6 (19)	2 (33)	4 (67)
Health concerns			
Child	7 (23)	3 (43)	4 (57)
Self	16 (52)	4 (25)	12 (75)
Language preference			
Spanish	18 (58)	6 (33)	12 (67)
English	13 (42)	2 (15)	11 (85)
Financial stability			
Work instability	21 (68)	7 (33)	14 (67)
Days worked per week			
5 days or less	10 (32)	3 (30)	7 (70)
6–7 days	21 (68)	5 (24)	16 (76)
Financial remittances	7 (23)	3 (43)	4 (57)
Off-farm income	7 (23)	0 (0)	7 (100)
Protective assets			
Grows food in a garden	22 (71)	6 (27)	6 (73)
Other safety net resources			
Informal food resources	11 (36)	5 (45)	6 (55)
Federal non-food programs	11 (36)	4 (36)	7 (64)
**Enabling**			
SNAP eligibility			
Income eligible^a^	8 (26)	4 (50)	4 (50)
Children <18	14 (45)	5 (36)	9 (64)
Disability	2 (6)	1 (50)	1 (50)
60+^b^	7 (23)	3 (43)	4 (57)
**Need**			
*Subjective food security*			
Adequate quantity	27 (87)	6 (22)	21 (78)
Meets food preferences	22 (71)	6 (27)	16 (73)
*USDA HFSSM evaluated food security*			
Food secure	18 (58)	5 (27)	13 (72)
Food insecure	13 (42)	3 (23)	10 (77)
**SNAP utilization**			
Currently utilizing SNAP	8 (26)	8 (100)	NA
Previously utilized SNAP	10 (32)	NA	10 (100)
Never utilized SNAP	13 (42)	NA	13 (100)

a Individuals who reported low income as the reason they are eligible for SNAP.

b As individuals aged 60+ are eligible to receive SNAP when net income limits are met, this characteristic is both predisposing and enabling.

Farm owners managed and operated small farms (farm income < $350,000), all but two of which owned the land they farmed on. Two farm owners operated family farms that had been passed down to them. Farm types included horticulture (polyculture, orchard, apiculture) and livestock. Most (*n* = 9) employed farmworkers at some point during the year.

### 3.2 Predisposing characteristics

Table 1 provides the differences in participant characteristics by SNAP program utilization, organized by variables adapted from the original Andersen model. Consistent with current statistics, higher proportions of SNAP utilization were reported among Hispanic/Latinx participants and those with children living in the home (48). In contrast to the literature, higher proportions of SNAP utilization were reported by participants who preferred to speak Spanish during their interview, those aged 60 and older, male, married, and that lived with immediate family only, compared to those who were not currently using SNAP. There were also higher proportions of SNAP utilization reported by farmworkers and farm owners who experienced work instability, those who worked five or fewer days per week, and individuals who provided financial support to family living in their country of origin (most non-U.S. born participants in the sample immigrated from Mexico). Regarding food acquisition strategies, SNAP users reported growing vegetables for home consumption, using informal food assistance like food pantries, and using federal aid programs that are not food-related (e.g., unemployment insurance) more frequently than individuals who did not use SNAP. Concerns about their children’s health and access to preferred foods but inadequate quantities of food were also reported more often among those who used SNAP. Among participants who used SNAP, most were food secure (63%).

### 3.3 An Andersen model of SNAP utilization on the farm

**Figure 1:**
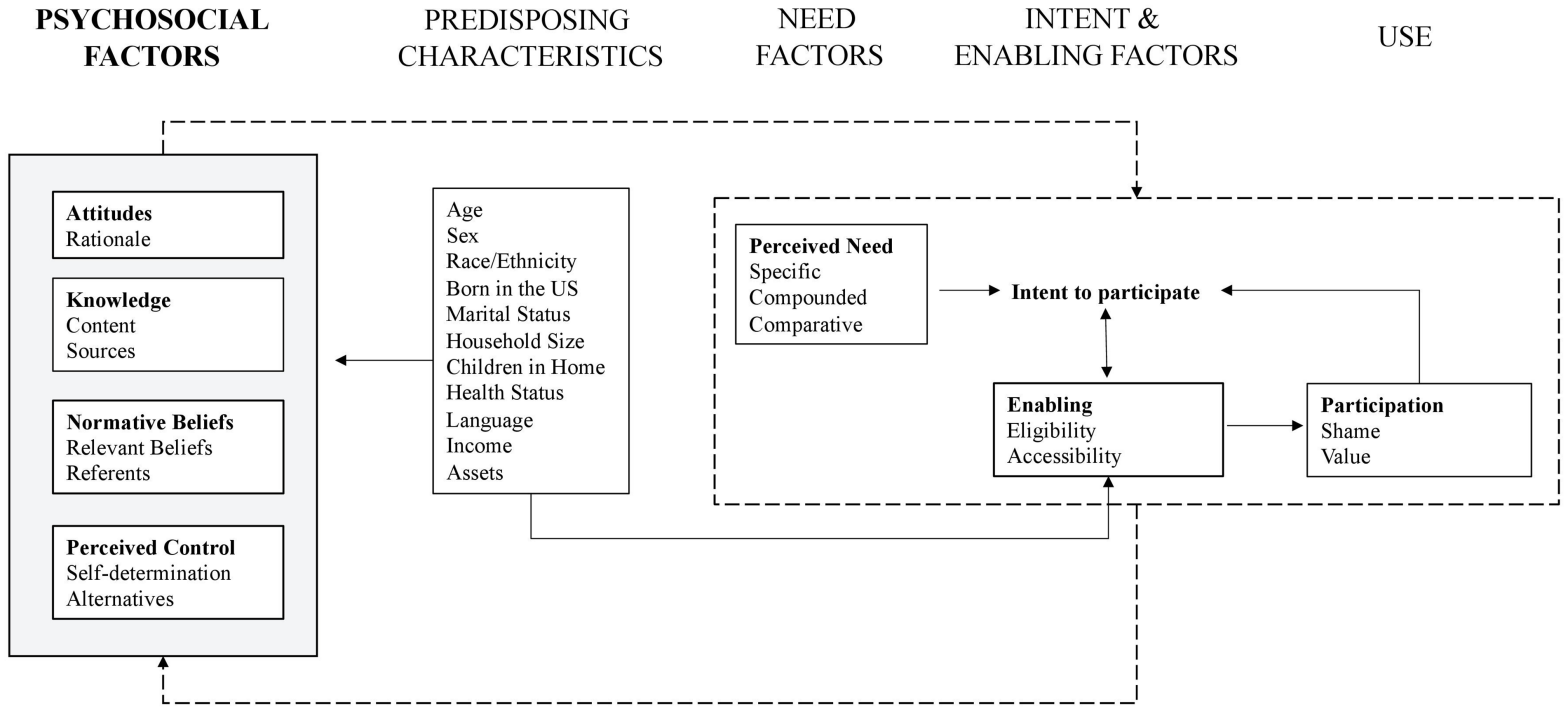
An Andersen model of SNAP utilization on the farm: adapted theoretical framework to depict the central role of psychosocial factors on need, enabling, and use factors in SNAP participation among farmworkers and farm owners in Oregon, USA.

The factors, domains, and themes identified in this study are summarized in Table 2, accompanied by select quotes. As the expanded Andersen model (34) and subsequent applications of it (49) have focused upon health service utilization, some elements were modified or shifted to capture the use of a public resource (SNAP) as opposed to a health service. The theoretical model connecting each element is depicted in Figure 1.

**Table 2:** Summary of factors, domains, themes, and select quotations of an Andersen model of SNAP utilization among farmworkers and farm owners in Oregon, USA.

Factor	Domain	Theme	Example quotes
Psychosocial	Attitudes	**Rationale**	“When out of nowhere, there is no work, and I do not have enough money… they gave us food stamps.” 56-year-old male farmworker who was using SNAP
		Justification for utilization	
		Temporary utilization	
	Knowledge	**Content of information**	“I think it’s a way to help people provide food for their families.” 42-year-old male farmworker who was using SNAP “Some people are in need [but] that’s why they have not requested food assistance [because they do not want to lose their green card]. It is hard.” 35-year-old male farmworker who was not using SNAP
		Program purpose	
		Intended audience	
		Supply of available resources	
		Eligibility & legal (public charge)	
		**Sources of information**	“I first heard about this program through my friends once I got injured.” 65-year-old male farmworkers who was using SNAP
		Friends	
		Community	
	Normative beliefs	**Relevant beliefs**	“We certainly qualify. [Shame associated with SNAP is] something that I try and unlearn, and I can unlearn for other people. I do not have any judgment of other people using them, but I just could not quite get there at that moment, myself.” 36-year-old female farm owner who was not using SNAP
		Stigma	
		Systematic program abuse	
		Welfare dependency association	
		**Referents**	“What they do not understand is that the work in the field is temporary.” 72-year-old male farmworker who was not using SNAP “There’s this Midwestern pride… My family–specifically I remember them just being really critical of people that use [SNAP]… it was like an ‘if they ever found out’ situation.” 36-year-old female farm owner who was not using SNAP
		Family	
		Community	
		Agency personnel/caseworker	
	Perceived control	**Self-determination**	**“**The only solution to [not having enough food] is, if for example, I do not have a job there, I go out to the field to look for work a day or two some place where they have work.” 72-year-old male farmworker who was not using SNAP “Do not go knocking on so-and-so’s house: ‘Hey (knocks on table) give me money to go buy tortillas or bread, chicken, beans …’ And that friend will say ‘That lazy [person] does not work …’.” 56-year-old male farmworker who was using SNAP
		Locus of control	
		Independence	
		Dignity	
		Privacy	
		**Alternatives**	“I have friends, friends that, if for any reason I do not have money for food. If I needed money, I can get some. If they need me and I can help them, I’ll gladly do it.” 72-year-old male farmworker who was not using SNAP
		Family support	
		Debt accumulation	
		Informal loans	
Need	Perceived need	**Specific need**	“After I pay my mortgage payment, I’m living on a couple of hundred dollars.” 65-year-old female farm owner who was not using SNAP
		Food security	
		Financial strain	
		**Compounded need**	“Sometimes my husband pays the rent and then sends money to his parents in Mexico and so on. Sometimes yes, sometimes we see difficult times, but saying that we do not have, that we do not have [money for food], no, no, no, no, no, we never completely run out of food. We always have some, for the next day.” 73-year-old female farmworker who was not using SNAP
		Competing hardship	
		**Comparative need**	“My [food] situation is not that serious. I have other concerns that are more serious… What can I tell you? Nothing in life is easy. All of the things you have to earn through hard work. The thing that damaged me the most were the work-related injuries.” 65-year-old male farmworker who was using SNAP
		Past hardship	
		Witnessed hardship	
Enabling	Availability of support	**Eligibility**	“We do not qualify for [SNAP]… because there’s only three of us at home… and we need to have a lower income. Because I do work during those [productive] months and I can cover the rent and the rest of the bills.” 52-year-old female farmworker who was not using SNAP
		Income	
		Eligible children	
		Disability	
		Age 60+	
		**Accessibility**	“They give you an appointment and you have to fill the paperwork and they ask you all sorts of things. And I really do not like it.” 44-year-old female farmworker who was not using SNAP
		Hassle	
		Agency personnel	
Use	Participation	**Shame**	“I felt bad buying quality food with my food stamps… I noticed little flashes of the eye between the checker and the bagger if I was using food stamps for certain things… I felt really kind of limited and almost like I needed to buy just like the cheapest of the cheap with my food stamps… you get like grilled when you use [SNAP]. Everyone behind you sees that you are using [them].” 40-year-old female farm owner who was not using SNAP
		Experienced stigma	
		**Value**	“They ask you how much money you make and if you are over the limit. And if you are over the limit by just a little bit you just get a few dollars. And for $50.00 or $100.00… I am not going to waste my time on that.” 44-year-old female farmworker who was not using SNAP “They’re more than generous. We can even get a steak once in a while if we want.” 75-year-old male farm owner who was using SNAP
		Adequacy of benefits allotted	

Predisposing characteristics (31) were aggregated with psychosocial health factors (34) to describe the elements that drive SNAP utilization for farmworkers and farm owners. Findings suggested that previous use of SNAP, “use factors,” influenced one’s intent to use SNAP in the future through an iterative process. In the following sections, we discuss these findings organized by factor, domain, and theme.

#### 3.3.1 Psychosocial factors

Psychosocial factors played the central role in determining SNAP utilization and included the following domains: attitudes, knowledge, normative beliefs, and perceived control.

##### 3.3.1.1 The attitudes domain

Participants’ attitudes influenced their rationale for SNAP utilization, including justifying need, “[The times] that we do not have [money] for food, they gave us food stamps… but it’s only when we really need them, not… I tell you, it’s only when there’s not a lot of work” (43-year-old male farmworker), and emphasizing the temporary nature of their use, “I had an accident, and I injured this leg. And [SNAP] helped me for a short while. Like one year and that’s all.” (58-year-old male farmworker).

##### 3.3.1.2 The knowledge domain

Knowledge of program purpose and audience (what SNAP is for and who gets to use it) jointly influenced views of program fit and alignment with participants’ specific set of needs (Figure 1). Individuals who believed the purpose of SNAP was to supply food for those without any would not utilize SNAP when they did not perceive an immediate need for food themselves: “I think the people [who] do not have food, if they qualify, they go to the stamps.” (73-year-old female farmworker). Whereas individuals who believed the purpose of SNAP was to provide aid for low-income individuals and families would utilize the program. A 54-year-old male farmworker explained: “These programs are given to those with low incomes… like us here. If you do not qualify, they will not give you anything.” (54-year-old male). The supply of available SNAP benefits also influenced the decision to participate. Concerns about exhausting a perceived limited resource led to feelings of guilt from farmworkers and farm owners who believed SNAP should be allocated to individuals with comparatively greater need: “I am somewhat relieved that I’m off [SNAP] because I felt so much guilt around it, and I always was like this really needs to go to someone who is like super down and out.” (40-year-old female farm owner).

Knowledge was acquired through friends, community members, and agency personnel as sources of information. For example, it was common for individuals to encounter hardship, like an injury or job loss, and be referred to apply for SNAP, “I first heard about this program through [several] friends once I got injured… And I applied immediately.” (65-year-old male farmworker). While referents sharing information related to public charge and SNAP use specifically centered around losing the authorization to live and work in the U.S. were not explicitly revealed, the feelings conveyed by some farmworkers suggested an atmosphere of collective fear that impeded SNAP utilization. For example:

*[There is] too much fear [in the community]. I know people who are needy and who receive food stamps during the winter season when we do not have too much work, and they were saying that [using SNAP] would affect them, that the green card would not be renewed, and those who are trying to obtain their documents will not be able to. It’s very tense. (35-year-old male farmworker)*

Concerns about public charge and U.S. residency were limited to farmworker participants.

##### 3.3.1.3 The normative beliefs domain

Normative beliefs were grounded in political stereotypes and stigmas, and shaped by family, community, and SNAP caseworkers. One farm owner who previously used SNAP recognized how stigma affected her utilization, “In my mind, my intelligent mind is like, yes. You should be using these food stamps for good, quality food, but I just had this weird social stigma around it.” (40-year-old female). Even among participants who conveyed that they did not hold stigmatizing beliefs toward others’ SNAP utilization, they still did not feel they could use SNAP because of the social stigmas attached to program use and the potential for judgment from others. Both groups related SNAP utilization to political narratives of program abuse and stereotypical welfare dependency. Farmworkers and farm owners shared beliefs that individuals do not need SNAP but use it anyway or that individuals who need to utilize SNAP are not diligent or must not work hard enough.

*Yes, there are people that I know do not need it. There are many people who do not work and get food stamps. These are just people who do not like to work, and they get food stamps. I mean, it’s obvious that they are going to get food stamps. I do not like that. I like to work, and I’d rather work to earn my money. (44-year-old female farmworker)*

For farmworkers and farm owners, who take pride in their identity as ingenuous hard workers, “You just go hard seven days a week, sun-up to sundown to make this farm happen.” (36-year-old female farm owner), the decision to use SNAP conflicted with their core identity.

Family, community, and SNAP caseworkers played a crucial role in perpetuating SNAP stigma. For some, stigma was common among their community, or it was intergenerational. For example, one 36-year-old female farm owner raised on a family farm discussed her inability to bring herself to apply for SNAP due to “a family history of shame.” Notably, another 31-year-old female farm owner who previously utilized SNAP after her husband lost his job mentioned a SNAP caseworker sharing narratives related to program abuse:

*The [caseworker] actually told him in a surprised voice, “I cannot believe that you are cutting this off – because so many people try to use it even after they do not need it” the guy was thankful [that my husband went in to discontinue SNAP].*

##### 3.3.1.4 The perceived control domain

Perceived control was characterized by self-determination and alternative safety nets. Though SNAP supported participants’ internal locus of control over their needs, it required them to relinquish control over how they are treated or view themselves. Most conveyed that it would be preferable and more dignified to maintain their food needs independently without using SNAP, but SNAP was better than having no access to food: “One thing that we were quite embarrassed to do, and we still are somewhat embarrassed because of the lack of income…we went ahead and got the [SNAP] card. So, we use that and it’s such a blessing.” (Farm owner couple in their 70’s).

One advantage of SNAP was the privacy it granted participants. One farmworker who was utilizing SNAP explained, “[It is better to] not bother other people … you can go to ask for stamps, right? It is confidential.” (56-year-old male). Farm owners appreciated the transition to electronic balance transfer (EBT) cards from physical food stamps, as it provided them more privacy at store checkout counters and helped preserve their dignity among small rural communities.

Despite the assistance that SNAP provides, some participants still experienced a shift in their locus of control. One comment highlighted the ways in which SNAP represented a loss of autonomy over food choice:

*I used to eat full meals and was able to decide what I wanted to eat. Now I can still decide, but it’s not the same… Because we took a big hit [so], I get [food] stamps. (65-year-old male farmworker)*

In lieu of SNAP, some farmworkers and farm owners opted to use alternative sources they associated with greater control over their food access. One farm owner who was relieved she no longer participated in SNAP explained how she made ends meet, “I have just been putting things on a credit card, so do I have the money for food? No, but do I have access to it? Yes.” (40-year-old female). Support from family, debt accumulation, and informal personal loans were common resources used to get by.

#### 3.3.2 Need factors

##### 3.3.2.1 The perceived need domain

Experiential and social inventory justified participants’ perceived need. Perceptions of need emerged in three ways: specific need, compounded need, and comparative need. Some farmworkers were prompted to use SNAP by the specific need for food. Over three quarters of farmworkers and farm owners in this sample experienced compounded needs and simultaneous burdens, which cued their intent to use SNAP. One farmworker described how SNAP helped when his household bills piled up, “Now I have to pay rent, electric bill, and other bills, so right now, we are getting food stamps.” (42-year-old male). One farm owner described how the compounded needs experienced among farming communities are more difficult to manage during the agricultural off-season:

*I might look into [SNAP] if, as the winter goes on and I’m really struggling, you just never know what kind of expenses. Two years ago, my horse had two abscessed feet. And between my dog that died and my horse being sick, the vet bills almost ruined me. So, I’ll just have to see. If it goes on [and] everything is okay, I’ll probably eek by this winter. But if I have any kind of major expense that comes up, I might be in there applying for food stamps. (63-year-old female)*

Participants’ comparisons of their current needs versus past needs or the needs of others, often deterred them from using SNAP. For farmworkers, difficult experiences during immigration and knowledge of the hardships endured in their country of origin, in this case, Mexico, sometimes overshadowed their current needs (i.e., it is not as bad now as it was then, or it is not as bad for me as it is for them). For example, one farmworker who was not using SNAP shared his assessment of comparative needs among his community, “I think that there aren’t as many people in need here [compared to Mexico]” (45-year-old male), highlighting how comparative need can challenge an individual’s capacity to appreciate their current needs provided comparatively better circumstances.

#### 3.3.3 Enabling factors

##### 3.3.3.1 The availability of support domain

Perceived SNAP eligibility was a barrier to some who wanted to participate but did not believe they qualify, “If I could, I would like to… get [SNAP].” (73-year-old female farmworker). The inconsistent, seasonal, and hazardous nature of farm labor made it difficult for many to meet SNAP eligibility rules, “what they do not understand is that the work in the field is temporary. Packaging [fruit] is temporary too.” (72-year-old male farmworker). This mismatch led to periods of eligibility and ineligibility throughout the year, and many participants were uncertain about their status. One farmworker explained how her husband’s work-related injury affected his ability to meet SNAP work eligibility rules:

*I was saying [to my husband], go and see if you qualify for [food] stamps… because you have not work[ed] for so long. But [because it had been so long since he worked] he does not qualify. (73-year-old female)*

Low income-based eligibility cutoffs and interim proof of eligibility checks frustrated participants who occasionally earned extra income from off-farm jobs or worked long hours over the growing season to arrive at a more sustainable income, only to result in a loss of SNAP benefits.

*Now that my husband is doing more construction and his focuses aren’t solely on the farm, we do not qualify for food stamps anymore, and that’s been really hard, too, because our main – I mean, we are just like incredulous every month. We are down to dollars in our account. (40-year-old female farm owner)*

Another theme that defined the availability of support domain was program accessibility, characterized by the experiences participants had applying or reapplying for SNAP and their encounters with caseworkers. Seasonality is just one issue the farm workforce encounters when applying or recertifying for SNAP. One farm owner described the hassle he encountered with providing proof of his self-employment on the farm.

*I was on food stamps. It was just, like, a hassle to apply again, or like, it’s kind of a unique situation with the farm. They think I wasn’t trying to find work or something. There was something where I had to find work as a fully employed [person], and I was just like, “[Expletive] it.” So, I did not even [apply again]. (36-year-old male)*

Many participants also raised challenges communicating with SNAP caseworkers who did not understand the unique fluctuating circumstances of farming communities, “I talk to [agency personnel] and then, they say to me, ‘Why do not you get a stable job?’” (72-year-old male farmworker), and an interrogative interview process that left them feeling undignified or disrespected. One farmworker who previously used SNAP explained how the effort of proving her need for assistance was not worth the benefits provided by SNAP:

*I used to get food stamps… they ask you all sorts of questions, and they want to know everything about you. You have to fill out so much paperwork and provide information about everything, and in the end, they just give you $50.00. To be honest, that is the reason why I no longer request them… If they are going to help us then they should just help you instead of telling you, “You have this much money. How much do you make? Why cannot you make ends meet?” (44-year-old female)*

#### 3.3.4 Use factors

##### 3.3.4.1 The participation domain

Perceptions of program value and experiences of shame directed future use. Lived experiences from previous SNAP utilization influenced one’s intent to use SNAP in the future through an iterative process in which experiences from use informed participants’ attitudes, knowledge, norms, and perceived control (psychosocial) and perceived hassle (enabling factors). Positive experiences were vital to determining intent to use and continued use of SNAP.

Feelings of shame were described exclusively by farm owners, and it affected the ways they used SNAP. One farming couple described the efforts they would go through to hide SNAP use from members of their small local community:

*We still feel a little [shameful using SNAP]. I used to take the [EBT card] out, and I kind of would not necessarily show what it was. When we first used it, we actually went to another little town to use it. (Farm owner couple in their 70’s)*

Another farm owner explained how her experiences with stigma using SNAP at the grocery store prompted her to end utilizing the program altogether.

*SNAP is great, but I would feel weird. I’m not kidding. I would see like looks. You know, like, someone picking up my can of, like, organic soup or something and… being like, “Oh, you are buying a $4.00 can of soup when you could buy a $2.00 [can of soup].” (40-year-old female)*

The value participants derived from their SNAP benefit allotment led to continued utilization. Those who were currently utilizing SNAP at the time of the study shared positive program feedback: “They’re more than generous.” (75-year-old male farm owner). Those who no longer participated in SNAP shared more critical program feedback: “To be honest, it’s not worth it.” (44-year-old female farmworker).

Some farm owners who did not use SNAP theorized about its potential value, but these thoughts did not necessarily motivate individuals to participate in SNAP, highlighting the importance of use here as an iterative factor within the theoretical model:

*[SNAP] would have been gratefully helpful to everybody on the farm, and then I would have gone to vendors at the farmers’ market… I could have been buying vegetables from some of my friends [to support their farm business] and maybe even getting [nutrition incentive vouchers] from the market to buy more vegetables. (36-year-old male)*

## 4 Discussion

Findings from this study revealed the central role that psychosocial factors played in determining farmworker and farm owner SNAP utilization. Program determinants were different from those found among the general SNAP population (30, 32). Three salient themes emerged, highlighting the ways in which broad federal policies may not reach uniquely vulnerable populations. Themes included varied needs, misalignment of need and eligibility, and pervasive internal and external stigmas associated with SNAP utilization.

Study participants’ nuanced perceptions of need played a role in their decision to utilize SNAP and their experiences while using the program. Types of perceived needs included specific, compounded, and comparative needs. Specific need indicated a lack of food. Most study participants who used SNAP reported that their food needs were not met, which aligns with research indicating that specific needs predict SNAP utilization among the general population (30). At the same time research documenting the association between SNAP use and greater food security (18) comports with the food secure farmworkers and farm owners who were using SNAP in this study. Notably, evaluated scores indicating food insecurity among those who were not using SNAP may be attributed to a loss of SNAP benefits by those who previously used the program, as the loss of SNAP has been associated with food insecurity (50). Farming communities experience multiple distinct vulnerabilities, such as immigration, isolated rural residency, harsh working conditions, and unpredictable and variable stressors, such as seasonal and market-related fluctuations in work availability, among others (8, 51). We found that SNAP may help alleviate some of the burdens rooted in compounded need among this chronically overextended and often low-income community. Despite these struggles, farmworker and farm owners’ appraisals of their comparative needs elicited feelings of guilt among some SNAP users and deterred others from using SNAP entirely. Such feelings occurred when study participants’ cognitive evaluations of need were based upon their prior hardships or struggles of others who may be worse off, paired with the belief that SNAP should only be distributed to those with the greatest need. As none of our study participants perceived themselves to be in great need, this resulted in a misalignment between their knowledge of the target audience for SNAP and how they identified with, or saw themselves as a member of, that audience.

Stigma and shame influenced the factors that determine SNAP utilization on multiple levels. A “culture of work” (52), rooted in hard labor, independence, autonomy, and self-sufficiency, was evident among the farm workforce who participated in this study. As such, stigmas that reflected associations of SNAP use with laziness and welfare dependency were particularly obstructive. Such external stigmas conflicted with participants’ self-identities as fastidious workers, prompting experiences of shame using SNAP and affecting their intent to participate. This finding is consistent with research showing that SNAP beneficiaries perceived as “less deserving” are more likely to internalize SNAP stigmas and experience diminished feelings of self-worth (53). Although one benefit of utilizing SNAP was its relatively confidential nature, some participants remained reluctant to use their benefits at their local grocery. SNAP users experienced intrapersonal stigma from store clerks, fellow shoppers, and SNAP caseworkers, which further disincentivized and hindered their access to SNAP. As caseworkers coordinate access to SNAP (enabling factors), the normative beliefs they perpetuate may be interpreted as fact rather than opinion, thus perpetuating the narrative of widespread abuse of SNAP or other safety net programs. These findings align with previous studies on the stigmatization of SNAP participants by the general population (53, 54).

### 4.1 Policy implications

Findings suggest the importance of balancing the needs of the farm workforce, benefits offered by SNAP use, and reluctance to utilize the program. Local and streamlined outreach on the farm and enrollment/re-enrollment efforts may help facilitate SNAP utilization among overburdened farming communities. As mobile medical clinics have successfully provided medical care among farming communities (55), a similar approach may provide a good model for SNAP personnel to engage locally and normalize SNAP use.

Program eligibility was a key driver of SNAP utilization. Consistent with the research, this study demonstrated that despite the needs that farming households may have, eligibility contingencies prevent them from utilizing the formal safety net to cope with food insecurity (27). Although need factors and psychosocial factors affect farmworker and farm owner intent to utilize SNAP, federal and state program eligibility guidelines must be satisfied to use the program. Among individuals who were eligible, more participated in SNAP than those who did not participate. Still, several farmworkers reported a desire to participate in SNAP but were unable to do so because of perceived or actual ineligibility. SNAP presumes a steady set of circumstances that farming does not fit. Employment-based eligibility criteria were particularly problematic, as they did not reflect the occupational inconsistency, seasonal fluctuations, or time off for work-related injuries common in farm employment (8, 13), leading to periods of eligibility and ineligibility throughout each year. In addition, income thresholds do not consider variable expenses that households may have. Immigrant households, for example, may have added costs of supporting family members living in their country of origin. Further, low eligibility thresholds cut off benefits to families whose incomes are just above the threshold but are nevertheless food insecure (56). Even among farmworkers and farm owners who were eligible for SNAP, the process of providing evidence of employment was a hassle or even impossible to prove among informal work agreements and self-employment. Public charge prompted other eligible farmworkers to discontinue program utilization for fear of losing legal residency within the U.S. Better alignment of SNAP eligibility in consideration of occupational circumstances, removal of interim proof of eligibility, and abolishing public charge rules could help bridge eligibility gaps among farming households.

Like the findings of the current study, stigma has been previously identified as a barrier to safety net utilization among low-income farming households (27). Policy initiatives directed at reducing shame and stigma are essential to support SNAP utilization. Community-engaged outreach mechanisms may help shift social norms around SNAP use. For example, it may be beneficial to recruit trusted members of the community to conduct preliminary screening for SNAP and connect individuals and families to services. Encounters with SNAP agency personnel may be improved by cultural competency and empathy skills training for staff and by hiring caseworkers who reflect the demographic characteristics and backgrounds of the eligible population, such as those who utilize SNAP themselves. In addition, mainstreaming remote case management, such as through online platforms that can be accessed with a smartphone, may reduce shame and stigma associated with interpersonal encounters.

### 4.2 Theoretical contributions

In this study of the farm workforce, we adapted the original and expanded Andersen models (31, 34) to create a theoretical framework that comprehensively captured the relationships among sociodemographic, psychosocial, cultural community-level factors, and SNAP utilization. To the authors’ knowledge, this study is the first to aggregate the original and expanded Andersen models toward an analysis of public assistance utilization. The model revealed the driving variables associated with the intent to and actual utilization of SNAP and mechanisms by which psychosocial, need, and enabling factors may be related. As potential access and realized access is signaled by enabling factors and utilization, the issues with SNAP eligibility, accessibility, and a low number of participants who were currently using SNAP suggests that farmworkers and farm owners experienced low access. Further, the central role of psychosocial factors in the model provides evidence of inequitable program access (31).

Adaptations from the original model included the intent to participate as a step toward utilization. In addition, a use factor was added to incorporate the experiences from actual SNAP utilization. As the original and expanded Andersen models are recursive (31, 34), the importance of use factors and depiction of the model as an iterative cycle, not a linear process, were essential to the proposed model. These findings suggest that SNAP utilization may mediate the effect of psychosocial determinants for future SNAP utilization through an iterative process. Understanding these relationships is fundamental to help identify appropriate strategies to address food insecurity among farming households.

### 4.3 Strengths and limitations

While this study is among the first to explore SNAP utilization among a sample of farmworkers and farm owners together, several limitations should be acknowledged. First, the present study analyzes data from a more extensive study using open-ended, semi-structured questions. Though the qualitative analysis was performed using an Andersen model framework, the study protocol was not designed explicitly for this purpose. Second, although many participants discussed needs, domains, and major themes related to their utilization of SNAP, the focus of the study protocol on food insecurity and coping strategies likely limited the depth of conversations related to SNAP. Finally, the farmworkers and farm owners in this study were not explicitly asked about the factors they considered when making the decision to use SNAP. As previously discussed, these factors enable the examination of the driving variables associated with the intent to utilize and actual utilization of SNAP and mechanisms by which psychosocial, need, and enabling factors may be related.

Second, the study sample included a limited sample of farm owners and farmworkers living in one state in the U.S., all farmworkers in this study were Hispanic/Latinx and Spanish speaking and recruited from one Oregon county, and most farm owners were White and English speaking. As such, these attributes may limit the transferability of these findings to farming communities in other regions of Oregon and other states. Further, interviews were conducted before the COVID-19 pandemic, which had a substantial impact on farming communities. These findings reflect pre-pandemic vulnerabilities and coping mechanisms, limiting the dependability of findings to the post-pandemic farm workforce. For example, the farm owner and farmworker role as essential workers during a global pandemic placed them at higher risk for exposure and transmission of COVID-19 (57). The work-related stigmas evidenced in this current study differ from stigmas related to farm work in field conditions that did not adhere to public health guidelines intended to limit the spread of COVID-19, like social distancing or proper use of personal protective equipment (58). While farm owners and farmworkers who could not work may have experienced shame related to the subsequent decrease in income and self-sufficiency, farm owners and farmworkers who could work may have experienced shame associated with the potential of spreading the deadly virus (59). Future research is needed to understand the role of SNAP among farming communities after the COVID-19 pandemic.

Lastly, two White women and one Puerto Rican Latina connected with a school of public health at a major public university conducted participant interviews. As with any research, the difference in gender, race/ethnicity, and social positioning between the interviewers and participants may have influenced the course and content of the interview. For example, participants’ comfort, openness, and responses may have been impacted by the social differences between the participants and the interviewers, which may have resulted in underreporting of SNAP utilization among participants.

To our knowledge, no studies to date specifically examine the factors that contribute to SNAP utilization among the farm workforce. A better understanding of the role of SNAP among farm owners and farmworkers enables the development of more responsive interventions to alleviate food insecurity for uniquely vulnerable farming communities. While this study contributes to our understanding of farm owner and farmworker decisions to utilize SNAP, further research is needed to document farming households’ experiences using SNAP and complementary social safety net programs to help cope with food insecurity. Future research would benefit from more rigorous sampling methods to test the relationships between psychosocial factors and SNAP use with greater precision. Additionally, future studies could test the strength of the adapted theoretical model used here to predict SNAP utilization among farmworkers or farm owners separately to understand both groups better and make distinctions between them. Finally, quantitative analysis of the predictive power of the sociodemographic, predisposing, enabling, and need factors would help determine the reasons some choose not to participate.

## 5 Conclusion

Gaps in SNAP utilization among farmworkers and farm owners highlights the need for evidence-based policy solutions. In this qualitative study of farmworkers and farm owners in Oregon, we found evidence of inequitable access to SNAP. Unique sociodemographic, psychosocial, and community level cultural factors help explain reasons for SNAP utilization or non-utilization specific to farming households. Moreover, this work highlights the importance of including use factors in applying Andersen model adaptations to safety net program utilization. These findings underscore the need for expanded SNAP eligibility cut-offs and targeted community engagement mechanisms to facilitate utilization.

## Data availability statement

The raw data supporting the conclusions of this article will be made available upon request to the authors, and without undue reservation.

## Ethics statement

This study involving humans was approved by the Oregon State University Institutional Review Board. The study was conducted in accordance with the local legislation and institutional requirements. Written informed consent to participate in this study was not required from the participants in accordance with the national legislation and the institutional requirements. Informed consent was obtained from the individual(s) for the publication of any potentially identifiable images or data included in this article.

## Author contributions

BR: Conceptualization, Data curation, Formal analysis, Investigation, Methodology, Project administration, Software, Visualization, Writing – original draft, Writing – review & editing. SG: Formal analysis, Funding acquisition, Resources, Supervision, Writing – review & editing. JG: Formal analysis, Writing – review & editing. ES: Writing – review & editing. MB: Writing – review & editing.
